# Male Lower Urinary Tract Dysfunction: An Underrepresented Endpoint in Toxicology Research

**DOI:** 10.3390/toxics10020089

**Published:** 2022-02-16

**Authors:** Nelson T. Peterson, Chad M. Vezina

**Affiliations:** 1 Molecular and Environmental Toxicology Graduate Program, University of Wisconsin-Madison, Madison, WI 53706, USA; ntpeterson3@wisc.edu; 2 Department of Comparative Biosciences, University of Wisconsin-Madison, Madison, WI 53706, USA

**Keywords:** lower urinary tract dysfunction, lower urinary tract symptoms, BPH, prostate

## Abstract

Lower urinary tract dysfunction (LUTD) is nearly ubiquitous in men of advancing age and exerts substantial physical, mental, social, and financial costs to society. While a large body of research is focused on the molecular, genetic, and epigenetic underpinnings of the disease, little research has been dedicated to the influence of environmental chemicals on disease initiation, progression, or severity. Despite a few recent studies indicating a potential developmental origin of male LUTD linked to chemical exposures in the womb, it remains a grossly understudied endpoint in toxicology research. Therefore, we direct this review to toxicologists who are considering male LUTD as a new aspect of chemical toxicity studies. We focus on the LUTD disease process in men, as well as in the male mouse as a leading research model. To introduce the disease process, we describe the physiology of the male lower urinary tract and the cellular composition of lower urinary tract tissues. We discuss known and suspected mechanisms of male LUTD and examples of environmental chemicals acting through these mechanisms to contribute to LUTD. We also describe mouse models of LUTD and endpoints to diagnose, characterize, and quantify LUTD in men and mice.

## 1. Introduction

LUTD is a deviation from normal urinary voiding. While LUTD occurs in males and females, disease mechanisms differ between sexes. The prostate plays a considerable role in male LUTD, the focus of this review. For such a pervasive disease, male LUTD has suffered from a surprising lack of research attention. Part of the problem is the disease’s complexity, driven by a constellation of underlying factors across multiple organs that are incompletely understood. Another problem is that the historical research record for LUTD is muddled by vast and inconsistent nomenclature used to describe the disease, decentralizing the resource of primary peer-reviewed literature. Several vocabulary terms are used to describe histological, anatomical, physiological, and clinical pathologies in the lower urinary tract. The following terms are sometimes conflated or interchanged with LUTD, and often used inappropriately: benign prostatic hyperplasia (BPH), benign prostatic enlargement (BPE), bladder outlet obstruction (BOO), partial bladder outlet obstruction (pBOO), lower urinary tract symptoms (LUTS), and others. These terms are defined in Table 1.

Male LUTD can be confirmed by specialized urodynamic studies at the urology clinic (diagnostic and experimental approaches used to identify LUTD mechanisms in mice and humans are described in Table 2). However, male LUTD is most often identified in the primary care clinic based on patient reported symptoms. LUTS can include but are not limited to weak stream, incomplete bladder emptying and more frequent voiding, especially at night. Male LUTD frequently begins in the fifth decade of life or later and is a progressive disease that can result in a loss of bladder function, bladder and kidney stones, acute urinary retention, and renal injury/failure [1,2,3,4,5,6,7]. LUTD disrupts sleep and has been linked to depression, decreased workplace productivity, and a reduced quality of life [8,9,10,11,12]. If not successfully managed, LUTD can be fatal.

LUTD is extremely common. A 2008 study estimated that 1.9 billion people, representing 45% of the population, are affected by LUTD [9]. The economic burden of LUTD is staggering. The disease affects more than half of men over 50 years of age in the Western world, resulting in $4 billion for the pharmacological treatment and $2 billion for the surgical treatment of LUTD and associated prostatic problems [13,14,15]. The most common therapies for male LUTD are directed to block alpha adrenoreceptor function (alpha blockers) and dihydrotestosterone synthesis (steroid 5 alpha reductase inhibitors), factors which contribute to prostatic smooth muscle contraction and prostatic enlargement, respectively. Unfortunately, these therapies are incompletely effective. Their magnitude of effect is marginal, not all patients respond, and existing therapies are only moderately protective against disease progression [16,17,18]. It is becoming clear that male LUTD derives from many different mechanisms, not all of which are addressed by current therapies. Factors responsible for severe drug-refractory disease are not understood. Recent studies reveal potential roles for environmental chemical exposures, during the fetal period when the lower urinary tract is developing [19,20,21] and during other stages, in driving LUTD susceptibility and progression, opening an entirely new line of toxicology research towards understanding environmental factors that contribute to LUTD processes.

This review is intended as a resource for toxicologists and other discipline specialists who are considering entry into the urologic disease research space and wishing to examine LUTD as a toxicology research endpoint. We describe the anatomy, cellular composition, and physiology of male lower urinary tract organs including the bladder, urethra, and prostate. We describe known and emerging disease mechanisms. We also highlight the limited examples of how environmental chemicals influence male LUTD and list opportunities for future research. 

## 2. Overview of Male Lower Urinary Tract Anatomy and Physiology

**Figure 1:**
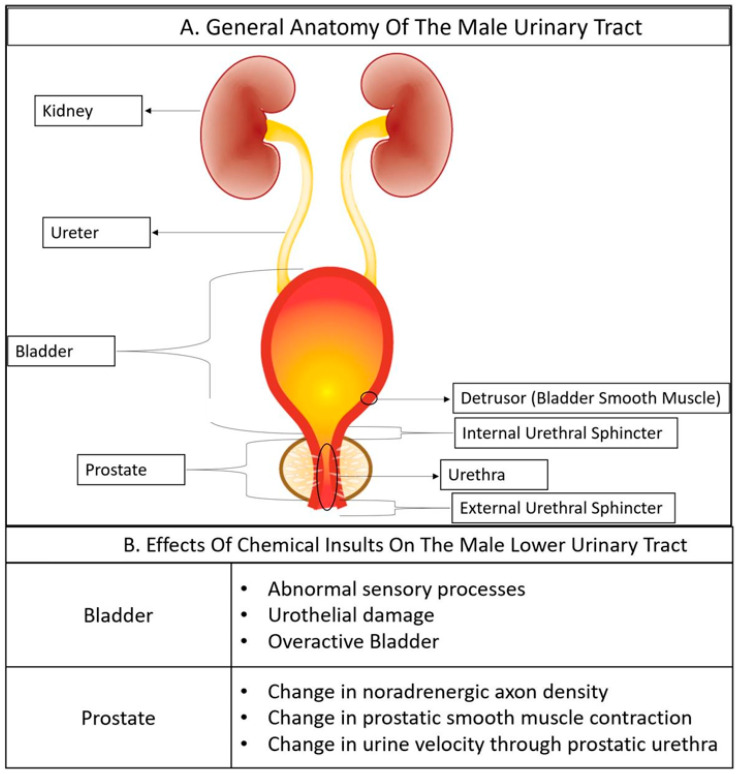
General anatomy of the male urinary tract and effects of chemical insults on the male lower urinary tract. (**A**) A general depiction of the male lower urinary tract. (**B**) Known effects of environmental chemicals on the lower urinary tract of either the man or male mouse.

Several benign diseases of the lower urinary tract are accompanied by a change in distribution, type, or state of cells that comprise lower urinary tract tissues [22,23,24]. Therefore, we describe the cellular anatomy of the male lower urinary tract to give toxicologists an appreciation of the normal cellular organization and changes which occur in response to chemical insults and disease The male lower urinary tract consists of the bladder, prostate, and urethra (Figure 1). Urine flows from the kidney to the bladder via the ureter and passes through the prostatic urethra and prostate before continuing through the penile urethra and exiting the body as voided urine (Figure 1). 

### 2.1. The Bladder

The bladder’s primary functions are to store and expel urine. The bladder wall consists of three tissue layers: a specialized epithelium known as the urothelium, the lamina propria, and the bladder smooth muscle (detrusor) [25,26].

The mature urothelium is comprised of basal, intermediate, and superficial cells [27]. Bladder epithelial cell differentiation begins early in fetal development (weeks 7–8 in humans), and the trajectory of urothelial cell differentiation during development and regeneration is susceptible to epigenetic modification [28] revealing a potential mechanism of toxicity for epigenetic modifying chemicals. The mature urothelium must achieve three unique functions. The first is to maintain distensibility to accommodate bladder filling and emptying. Bladder volume increases significantly during the storage cycle, a process which would normally challenge the integrity of an epithelial lining [29]. Bladder distensibility is achieved by urothelial cell junction rearrangements and cell sliding during bladder filling [29]. 

The second role of the urothelium is to protect sub-urothelial tissue from toxins, microorganisms, and urine solutes [27,30]. Barrier function is facilitated by secreted uroplakins [31]. Uroplakins are transmembrane proteins which assemble into a crystalline structure and are interrupted by hinge regions to allow bladder distension [32]. Uroplakins assemble to form uroplaques, rigid bio-membrane structures which cover 90% of bladder lumen [32]. Uroplaques are integral to the integrity, flexibility, and solubility of the urothelium [33]. The control of urothelial cell division is integral to maintaining functional uroplaques and restoring them after bladder damage. Although the urothelial cell turnover is normally slow with a labeling index of 1% in mice, the urothelium is reconstituted quickly after injury through the progenitor activities of basal and intermediate cells [32,33,34,35,36]. The epithelium of the urothelium can be completely repaired in 4 weeks in guinea pigs and 6 weeks in men [36]. Some mechanisms by which the bladder restores barrier function are surprising. For example, we found that under certain circumstances when widespread urothelial cell death depletes the bladder of its own progenitors, it can recruit non-resident, non-bladder (Wolffian duct) epithelial progenitors, drive their differentiation into uroplakin secreting superficial cells and restore barrier function [37]. Barrier function is crucial because sub-epithelial bladder cells are severely compromised by urine exposure. The experimental use of cyclophosphamide, an antineoplastic used therapeutically for Hodgkin’s lymphoma, multiple myeloma, and other cancers, has widened the understanding of barrier function and consequences of barrier function loss. Cyclophosphamide is bio-transformed into acrolein, which accumulates in the urine and drives urothelial cell death, resulting in hemorrhagic cystitis and changes in physiology [38,39,40]. Environmental chemicals with urothelial cell toxicity are expected to drive bladder inflammation and dysfunction like that of cyclophosphamide.

The third role of the urothelium is that of a sensor. In combination with nerve terminals within the bladder, the urothelium detects and responds to mechanical and chemical stimuli to alter detrusor contractility and moderate bladder afferent nerve activity [41,42]. Factors released by urothelial cells include acetylcholine, adenosine triphosphate (ATP), nerve growth factor, nitric oxide (NO), prostaglandins, and others [41,43].

The lamina propria contains a fibroelastic connective tissue with intervening afferent and efferent nerve fibers, a vast vascular network and dispersed fibroblasts, a loose smooth muscle layer (the muscularis mucosa), and myofibroblasts [26,44]. The elastic fibers within the lamina propria allow the bladder to recover its original shape after voiding [45]. 

The detrusor is the major smooth muscle component of the bladder [46]. The detrusor is organized as a circular muscle inner layer sandwiched between longitudinal muscle outer layers [46]. Muscle bundles are surrounded by collagen [46,47,48]. Detrusor contraction is predominantly controlled by cholinergic neurons [49,50], but can also be induced by purinergic neurons and relatively rare sympathetic neurons [49,50].

The normal voiding cycle is divided into filling and voiding phases [51]. Urine expands the bladder during the filling phase, while bladder pressure remains lower than urethral pressure [50,51]. There is still uncertainty about how the bladder relays the perception of fullness to the brain. One possibility is that mechanoreceptors and mechanosensitive ion channels within the bladder transmit information about fullness to afferent neurons [52,53,54,55,56]. There is also evidence that urothelial cells, stretched during bladder filling, release ATP to activate purinergic receptors on bladder afferents and relay bladder fullness to the brain [57,58,59]. Another possibility is that the perception of fullness is not driven by a slow increase in bladder pressure (intravesicular pressure), but rather by an increasing rate of spontaneous transient contractions, also called micromotions, which exist throughout the filling phase. Micromotions drive the major portion of afferent outflow to the central nervous system during bladder filling, acting in part through a mechanism involving calcium-activated potassium (SK type) channels [60].

In 1925, F.J.F. Barrington identified a brain stem region which controls micturition, including sensation of bladder fullness and the contractions leading to urination [61]. Studies using retrograde and anterograde neuronal labeling pinpointed the location of this micturition center in the pontine tegmentum [62,63,64,65,66,67]. This site of micturition control is referred to as Barrington’s nucleus, the pontine micturition center, and the M-region [62]. Afferent and efferent urinary voiding pathways are integrated in Barrington’s nucleus. During the storage phase, glutamatergic neurons in the periaqueductal gray and hypothalamus relay information about bladder fullness and bladder volume threshold for voiding to Barrington’s nucleus [68,69]. During the voiding phase, corticotropin releasing hormone-positive and estrogen receptor 1-positive neurons within Barrington’s nucleus activate efferent pathways to drive detrusor contraction [62,70,71]. Additional neurons in Barrington’s nucleus send inhibitory signals to the external urethral sphincter, driving its relaxation and allowing urine to flow unimpeded from the bladder into the urethra [71,72,73]. Though there is widespread evidence that environmental contaminants can disrupt connectivity, complexity, arborization, and signaling of neurons within the peripheral and central nervous system, whether environmental chemicals impact bladder ascending and descending neural pathways is rarely examined [74,75,76,77,78,79,80].

There is limited evidence that environmental chemical exposures can disrupt bladder neural circuitry as it is established during the fetal and neonatal periods, raising concerns about a developmental basis of bladder health and disease. A recent study tested the impact of exposure to a polychlorinated biphenyl (PCB) mixture on bladder structure and function [19]. The PCB mixture used in this study mimics the most encountered congeners in women who are at risk for having a child with a neurodevelopmental disorder [81,82]. PCBs were delivered orally to nulliparous female mice (75% C57BL/6J/25% SVJ129) starting two weeks before mating, through pregnancy and lactation, and continuing in offspring before their bladders were analyzed at postnatal days 28–31. The PCB mixture increased densities of sub-urothelial beta-3 tubulin (general neural fiber marker) fibers and calcitonin gene-related peptide positive (peptidergic fiber marker) fibers in male mice but not female mice, and these changes were accompanied by an increase in male bladder volume [19], suggesting they were sufficient to drive a change in bladder function. 

### 2.2. The Urethra

The human male urethra is divided into two parts, consisting of five segments: the anterior urethra (fossa, penile, and bulbar segments) and the posterior urethra (membranous and prostatic segments) [83]. The rodent male urethra is divided into two parts—penile and pelvic [84]. The human and rodent urethra are populated by epithelial cells, smooth and striated muscle cells, blood vessels, and sensory and motor neurons [85]. While the cellular components of the anterior/penile urethra have not been extensively characterized, single cell ribonucleic acid (RNA) sequencing approaches have been used to determine the cellular components of the prostatic urethra [24,86,87]. Urethral epithelium consists of club cells, hillock cells, basal epithelial cells, and neuroendocrine cells [86]. Urethral club and hillock cells were recently identified, but their functional characterization is incomplete and represents a future research opportunity. Lung club cells, which are transcriptionally like those in the urethra, act as progenitors and mediate anti-inflammatory and antioxidant processes [88,89,90]. Lung hillock cells, which are transcriptionally like those in the urethra, serve as progenitors, and participate in barrier function and immunomodulation [91,92]. 

### 2.3. The Urethral Sphincter

The urethral sphincter serves as a valve to regulate urine flow between the bladder and urethra [93]. During the bladder storage phase, urethral pressure exceeds bladder pressure to maintain continence [50]. During the voiding phase, the urethral sphincter falls open, urethral pressure decreases while bladder pressure increases, the urethra distends and urine flows through the prostatic urethra and penile urethra to become voided urine [50]. The urethral sphincter is divided into two parts: the external sphincter and the internal urethral sphincter [93,94]. The external sphincter consists of striated muscle circumscribing the urethra and is under voluntary control [93,95]. The internal urethral sphincter is indistinct from the rest of the lower urinary tract smooth muscle (bladder smooth muscle is continuous with urethral and prostatic smooth muscle), but is physiologically defined by its autonomic regulation, connected via a reflex arc to the bladder [95,96]. Urethral smooth muscle is organized as a thin longitudinal superficial layer, a dense circular layer, and a thin longitudinal deep layer [94].

### 2.4. The Prostate

The prostate synthesizes a portion of the ejaculate [97]. Prostatic smooth muscle contracts during ejaculation to propel prostatic fluid into the urethra [98]. The prostatic urethra also distends to accommodate urine during voiding. Benign prostatic disease changes the prostate’s histology and cellular composition and can prevent prostatic urethral distention during voiding, causing BOO, a common etiology for LUTD (defined in Table 1).

The human prostate is a spherical gland encapsulated by a fibromuscular sheath known as the prostatic capsule [24,96,99]. The base of the prostate is adjacent to the bladder and the prostatic urethra courses through its center [100]. The prostatic ductal network is like that of a branched tree: the main ducts drain directly into the urethra and divide into primary, secondary, and tertiary branches as they extend towards acini concentrated in the gland’s periphery [101]. The human prostate is organized into zones, differing in cellular composition and responsiveness to disease, and includes the transition zone (most susceptible to histological BPH, defined in Table 1) [24,102], the central zone and the peripheral zone (most susceptible to prostate cancer) [100,102]. The rodent prostate, often used as a disease model for humans, is anatomically distinct from the human prostate in that it is not spherical, but instead divided into four bilaterally symmetrical lobes: the anterior, dorsal, lateral, and ventral prostate [102]. While spontaneous cancer is not observed in the mouse prostate, a variety of genetically engineered mouse models are susceptible to prostate cancer and disease incidence differs by lobe [103]. The mouse prostate gland develops BPH spontaneously with age, but lesions are diffuse, like those that contribute to clinical disease in the dog, and unlike nodular BPH in the humans [104,105]. The rodent prostate ductal network is organized as a branched tree, like that of the human prostate, but ducts are surrounded by a looser stroma than in human prostate and the rodent gland is encapsulated in a thin adventitia instead of the thick capsule that surrounds the human prostate.

Human prostatic epithelium is made up of luminal, basal cells; neuroendocrine, club and hillock cells are also present, but are rare in prostate compared to urethral epithelium [22,86,106]. Human prostate stroma consists of three smooth muscle cell types (peri-prostatic, vascular smooth muscle and pericyte), two fibroblast cell types (peri-epithelial and interstitial), leukocytes, endothelial cells, and sensory and autonomic nerve fibers [86]. Mouse prostate stroma contains three fibroblast cell subtypes distributed in distinct proximal–distal and lobe-specific patterns and smooth muscle [24,106]. The transcriptomes of mouse prostatic and urethral fibroblasts are like human interstitial fibroblasts [24]. However, mouse urethral and ductal fibroblasts evoke Wingless related-integration site (Wnt) and Transforming growth factor beta (TGFβ) signaling pathways that are less abundant in human prostate fibroblasts [24]. Human peri-epithelial fibroblasts instead express Wnt inhibitors that could buffer Wnt ligands produced by other stromal or epithelial cells [24]. Human prostatic fibroblasts are organized in layers that center around epithelial structures, while mouse prostatic fibroblasts are not layered and differ by lobe [24]. Human and mouse prostate fibroblasts are most abundant in the proximal regions of prostatic ducts and least abundant in acini in the distal regions [24,107]. 

The recent observation, derived from single cell RNA-sequencing data, that human and mouse prostate cellular landscapes are similar, is also supported by previous microarray data [108]. Similarly, mouse prostate organogenesis is like that of the human prostate [109]. These data support the use of mice as a relevant model species for studying cellular and molecular mechanisms of benign prostatic disease. The key to understanding the differences in prostate architecture and benign prostate hyperplasia manifestation between these species may lie in the function of the specialized prostate epithelial and stromal cells of these species [109]. 

Prostate disease can be detected by changes in the spatial distribution and frequency of prostate cells [24]. Prostate cell immunophenotyping has proven difficult, as disease processes frequently lead to changes in cell state and cell type that cannot be easily distinguished by simple immunohistochemical staining protocols. New and validated RNA-Sequencing approaches, as well as cell sorting protocols deriving from them, have recently been described [86] and will be essential for elucidating prostate cell functions in future studies. 

## 3. LUTD Mechanisms

### 3.1. Benign Prostatic Diseases 

A variety of benign prostatic conditions contribute to male LUTD, many of which are believed to cause LUTD by driving BOO (defined in Table 1). The impacts of BOO extend beyond the prostate and into the bladder. A prolonged intravesicular pressure increase and bladder contraction against resistance reprograms the bladder in a process known as bladder compensation: the detrusor becomes thicker [110], it undergoes functional changes in ion channel physiology [111] and efferent signaling is reprogrammed [112]. If BOO is not effectively addressed, the bladder decompensates, much like a heart undergoing hypertrophic cardiomyopathy: the detrusor thins, is replaced by fibrotic tissue, and becomes incapable of mounting an effective contraction to fully evacuate urine from the bladder. There is evidence in rabbits that bladder decompensation is at least partially reversed by relief of bladder outlet obstruction [113]. Recovery from BOO likely depends on the severity of bladder decompensation at the time of surgery [113,114,115]. Thus, BOO must be effectively addressed before it permanently impairs bladder function. 

BPH is a leading cause of LUTD in men of advancing age. Human BPH is defined by prostate histology, specifically the presence of stromal, epithelial, or mixed nodules in the central and transition zones (Table 1) [22,116,117,118,119,120]. Small hyperplastic nodules can form as early as the 3rd decade of life and increase in frequency and volume with advancing age [121]. BPH mechanisms are not fully understood, but it has been hypothesized that BPH arises from a reawakening of embryonic signaling pathways [121] or disrupted homeostatic regulation of cell growth and death programs [116,117,118,119,120]. 

Aging-related changes in circulating testosterone and 17-beta-estradiol concentrations are another mechanism linked to male LUTD. Serum and prostate tissue concentrations of testosterone and 17-beta-estradiol change with age in men [122,123] and the changes are associated temporally and mechanistically with male LUTD [124,125,126]. Pharmacological alterations in testosterone and 17-beta-estradiol are a proven cause of LUTD in non-human male primates, canines, rats, and mice [124,127,128,129,130,131,132,133]. In mice, slow-release implants of testosterone and estradiol drive an increase in voiding frequency, a reduction in voided volume, an increase in collagen deposition, and a change in velocity of urine flow through the prostatic urethra [124]. The mechanism by which changes in circulating testosterone and 17-beta-estradiol drive voiding dysfunction are not clear but may include direct actions on the bladder [134,135], changes in prostatic desmin and smooth muscle actin content or function [136,137,138,139]. 

The fact that LUTD arises from natural changes in circulating sex hormone concentration raises questions about impacts of endocrine disrupting chemicals on male voiding function, and this area of toxicology research is in its infancy. For example, subcutaneous implants of the estrogenic chemical bisphenol A (BPA, 25 mg), combined with testosterone (2.5 mg) and given to C57BL/6N adult (6–8 weeks old) male mice, increase bladder mass and volume, increase voiding frequency, and reduce the volume of voided urine, suggestive of BOO [140]. BPA may act more broadly in the lower urinary tract, affecting the bladder as well as the prostate. Delivery of BPA (0.05–0.5 mg/kg/day) to Pietrain × Duroc mixed-breed juvenile female pigs increases the number and thickness of vasoactive intestinal polypeptide (VIP) expressing neurons in the bladder wall [141], raising questions about the influence of BPA, and the larger class of environmental estrogens to which it belongs, on detrusor recovery after contraction. 

Prostate inflammation, also called prostatitis (defined in Table 1), is extremely common and has been closely associated with LUTD. Approximately 50% of prostate biopsy, surgical or autopsy specimens harbor evidence of histological inflammation, most typically characterized as chronic (lymphocytic) inflammation [142]. The incidence of prostate histological inflammation is even higher (75%) in men with LUTD [143]. The presence of prostate inflammation in a biopsy specimen correlates with risk of symptomatic progression, urinary retention, and need for surgery [142,144,145,146]. A significant proportion of men with histologically defined prostate inflammation will develop urinary dysfunction [147]. Two placebo-controlled drug trials, Reduction by Dutasteride of Prostate Cancer Events (REDUCE) and Medical Therapy of Prostatic Symptoms (MTOPS), correlate histological prostate inflammation in human male prostate with increased prostate volume [144]. MTOPS study outcomes reveal that men with histological inflammation are more likely to progress to advanced LUTD, including acute urinary retention [144]. A separate study found that men with prostatitis were 2.4 times more likely to develop BPH and the presence of histological prostate inflammation in baseline biopsies was associated with 70% increased odds of requiring later treatment for LUTD [146]. Despite clear evidence that some environmental chemicals can drive inflammation and modulate autoimmunity, there is little information about environmental impacts on prostate inflammation and this represents a future opportunity that can be examined using immunohistochemical and physiological methods in Table 2. 

There is a distinction between histological and clinical prostatitis: histological prostatitis is identified in histological tissue sections, while clinical prostatitis is diagnosed by physical examination, urinalysis, imaging, cystoscopy, or patient questionnaire (for example, The National Institute of Health Chronic Prostatitis Symptom Index (NIH-CPSI)) [148]. Clinical prostatitis accounts for a significant proportion of outpatient visits [149]. Clinical prostatitis includes acute and chronic bacterial prostatitis, nonbacterial prostatitis, and asymptomatic prostatitis [148]. 

Prostate fibrosis is a recently identified mechanism of male LUTD. Fibrosis is an abnormal, detrimental version of the wound-healing process and is characterized by collagen deposition and tissue stiffening [150]. Macoska et al. [151] were the first to report fibrosis in the human prostate and link collagen accumulation to tissue stiffness and LUTS severity. Subsequent reports linked prostate fibrosis to histological inflammation, LUTS, and resistance to a combination therapy of alpha blockers and 5 alpha reductase inhibitors [150,152]. Prostatic fibrosis is an evolutionarily conserved LUTD process, supported by the fact that collagens also accumulate within the prostates of aging intact dogs and mice [104,153]. Though triggers for prostate fibrosis are not fully known, and whether environmental contaminants drive prostate fibrosis has not been studied, prostatic fibrosis results from prostate inflammation secondary to *E. coli* infection or obesity in mice [154,155].

Prostatic smooth muscle dysfunction is the target of the most prescribed drug class for male LUTD, the alpha blockers, and can be studied experimentally using calcium flux assays and isometric contractility assays described in Table 2. A study by Baumgarten et al. [156] was the first to identify noradrenergic axons in the human prostate, a surprising discovery considering that autonomic outflow to the bladder is mediated instead by cholinergic axons. Receptor binding studies and isometric contractility assays showed that noradrenergic receptors in prostatic smooth muscle mediate prostate tissue contractility [157,158]. The outcomes of these studies ushered the hypothesis that prostatic smooth muscle hyperactivity impairs urine flow through the prostatic urethra to cause BOO in some men. While this hypothesis was the basis for developing alpha blockers for male LUTD, little research has been directed at identifying mechanisms of prostatic smooth muscle dysfunction, most notably dysfunction mediated by environmental chemicals. This area remains ripe for scientific exploration. Prostatic smooth muscle contraction is controlled by autonomic neurons and aging is one factor that may contribute to changes in prostatic innervation [124,159]. There is emerging evidence that environmental chemicals can also change prostatic innervation to cause prostatic smooth muscle dysfunction, specifically by acting during the fetal and neonatal periods when prostate autonomic innervation is established. For example, we recently showed in C57BL/6J mice that gestational exposure to the widespread environmental contaminant TCDD (a single 1 µg/kg oral maternal dose on the 13th day of gestation) increases noradrenergic fiber density (nerve terminals) in the prostate of male mouse fetuses without changing the density of cholinergic or peptidergic fibers [160]. TCDD-induced prostatic noradrenergic hyperinnervation persists into adulthood and is coupled to hyperactivity of prostatic smooth muscle and abnormal urinary function in mice, including increased urinary frequency [160]. These findings are important because they support the concept that prostate neuroanatomical development is malleable, at least in mice, and that intrauterine chemical exposures can permanently reprogram prostate neuromuscular function to cause male LUTD in adulthood. In contrast, exposure to TCDD and other aryl hydrocarbon receptor agonists during adulthood appear to protect against BPH in men [161,162]. Differing consequences of aryl hydrocarbon receptor activation in the fetal period, versus adulthood, highlight the need to control for age in studies that examine potential impacts of environmental chemicals on urinary function and LUTD.

### 3.2. Bladder Mechanisms of Male LUTD

A variety of bladder conditions can lead to urinary dysfunction. This section describes the most common causes of male LUTD.

Overactive bladder is characterized by involuntary detrusor contraction. Consistent changes in animal models of overactive bladder include patchy denervation of the bladder, enlarged sensory neurons, hypertrophic dorsal root ganglia, and an enhanced spinal micturition reflex [163]. Overactive bladder is often characterized by sensory dysfunction [163]. There is a role for muscarinic M2 receptors in the severity of urinary urgency [163]. Some individuals with overactive bladder have a thicker bladder wall, suggesting overactive bladder may derive from BOO in some men [163,164]. 

The etiology of overactive bladder is multifactorial, deriving from three major mechanisms: myogenic factors, urotheliogenic factors, and neurogenic factors [41]. Myogenic factors contributing to overactive bladder include spontaneous detrusor contractions in response to bladder distension, ischemia, and changes in smooth muscle properties over time [41]. Neurogenic factors may include abnormal sensory processes, abnormal afferent excitability, or in some cases, damage, or abnormalities in central processing [41]. Dimethylaminopropionitrile, used in the manufacture of polyurethane, is an inhalation hazard that acts through a neurogenic mechanism to cause overactive bladder [165]. Methyl mercury also causes overactive bladder through what appears to be a neurogenic mechanism [166,167]. Damage to the urothelium can also cause overactive bladder, as rupture of urothelial cells releases factors that can drive detrusor contractility and micturition [41]. Biphenyl, used as a resin, a heat transfer medium, and an anti-fungal, is an example of an environmental chemical that causes urothelial cell damage and death [168].

Underactive bladder, also known as detrusor underactivity, is defined by detrusor contraction of inadequate strength, and results in prolonged or incomplete bladder emptying [169]. Patients with underactive bladder have a diminished sense of bladder fullness and are unable to mount forceful bladder contractions [170]. Underactive bladder can occur after episodic overactive bladder, reminiscent of bladder decompensation after BOO. In fact, there is a documented relationship between LUTD, underactive bladder, and fibrosis of the bladder [171]. The interstitial cells of Cajal, a specialized cell population with smooth muscle pacemaking activity, have been implicated in underactive bladder. The frequency of interstitial cells of Cajal is reduced in mice with underactive bladder and is associated with reduced frequency and amplitude of detrusor contraction [172]. Rats driven by bladder outlet obstruction to develop underactive bladder are deficient in stem cell factor, a ligand for the receptor C-kit which controls proliferation and function of interstitial cells of Cajal, and an increase in stem cell factor restores detrusor contractility [172].

### 3.3. Urethral Mechanisms of Male LUTD

Detrusor sphincter dyssynergia is characterized by simultaneous contraction of the detrusor and urinary sphincter, thereby impairing urine outflow from the bladder [173]. Detrusor sphincter dyssynergia manifests in three distinct phenotypes: (Type 1) increased sphincter activity during detrusor contraction which then ceases, resulting in delayed urination, (Type 2) intermittent clonic contractions during voiding, resulting in intermittent stream, (Type 3) continuous sphincter activity during detrusor contraction, resulting in impaired voiding [174]. Detrusor sphincter dyssynergia is common in men with spinal cord injuries or multiple sclerosis and has the capability to drive bladder decompensation, elevate pressure in the ureter and pelvis, and cause hydronephrosis, renal scarring and terminal kidney failure [173,174].

Neurological disease commonly manifests in bladder dysfunction [175]. Autonomic nervous system lesions (stroke, tumor, traumatic spinal cord injury, myelopathies due to cervico-arthrosis spina bifida), disseminated lesions (Parkinson’s disease, brain trauma, multiple sclerosis, meningo-encephalitis,) and peripheral neuropathies (diabetes mellitius) have all been identified as mechanisms of bladder dysfunction [175] and act in part by disrupting coordination between the detrusor, urinary sphincter, and central nervous system [173,174]. While there are many examples of environmental chemicals causing neuropathies, the consequences on lower urinary tract function are rarely examined.

### 3.4. The Relationship between LUTD and Comorbidities

Recent studies connect LUTD to other diseases. For example, people with cardiovascular disease, diabetes and obstructive sleep apnea are at increased risk of developing LUTD [176,177,178,179]. A common thread linking these diseases is a change in hemodynamics connected to ischemic injury [180,181,182], a factor that independently drives LUTD in mice [174]. Environmental chemical exposures have been linked to cardiovascular disease and diabetes [183,184,185,186], and this is another mechanism by which they may drive LUTD. 

## 4. Mouse Research Models of Male LUTD

Here we describe animal models used to study various etiologies of LUTD. While it is important to realize that results from animal models are not always transferable to humans, it is also crucial to highlight that animal models are used in preclinical trails to test the safety and efficacy of drugs and are an invaluable tool to use in toxicological studies.

### 4.1. Benign Prostatic Hyperplasia

A variety of genetically engineered mouse models have been used to drive expression of growth factors or mitogenic hormones in the prostate. Androgen responsive promoter sequences, androgen-induced cre recombinase or viral promoters are used to target genetic modifications to mouse prostate tissue and overexpress fibroblast growth factor 2 or fibroblast growth factor 3 to drive epithelial BPH [187,188,189], overexpress prolactin [190,191,192,193] or interleukin 1 alpha [194] to drive epithelial and stromal BPH and prostate inflammation, delete serine/threonine kinase 11 to promote stromal BPH in the periurethral region [195], or genetically modify other sequences. Expression of an activated form of P110 alpha, the catalytic subunit of PI3K, in mouse prostate epithelium also drives epithelial BPH in mice but accompanied with a stark fibrotic response in prostatic stroma [196]. Many of these genetically engineered mouse models were created before contemporary methods were optimized for mouse urinary physiology phenotyping. The historical goal was to use genetically engineered mice to identify molecular mediators of BPH and test efficacy of drugs and dietary substances for relieving BPH in preclinical model species. While genetically engineered mouse models are useful for understanding homeostatic mechanisms of prostate cell proliferation, it is becoming clear that BPH is not always linked to LUTD in men [197], and it remains important to characterize urinary physiology in these mice as a more relevant endpoint for male LUTD.

### 4.2. Mouse Models of Prostate Inflammation

Histological inflammation of the human prostate is extremely common: in one study, it was detected in nearly 80% of prostate biopsy specimens from 60+ year old men and was strongly associated with urinary voiding symptoms [198]. Prostate infection by ascending microbes is one potential mechanism of prostate inflammation and supported by the frequent encounter of bacteria in human prostate tissue specimens [199,200,201]. One strategy for driving prostate inflammation in mice involves urethral catheterization and delivery of uropathogenic *E. coli*. A variety of isolates have been used (*E. coli* UTI89, 4017, 1677 and CP-1), ranging from those collected as clinical urine isolates from women with bladder infections, to others collected from men with pelvic pain [202,203]. The pattern of inflammation (acute vs. chronic) depends in part on mouse strain used [204] and method of *E. coli* delivery (single vs. multiple inoculations, catheter size, instillation volume and bacterial load). It is essential to control these variables carefully when considering experimental design, and mice instilled with sterile saline (sham operated mice) are an essential component of experimental design because urethral catheterization can itself induce trauma, urethritis, and changes in urinary voiding physiology [144]. Prostatic *E. coli* infection is linked to prostate fibrosis and changes in voiding patterns in mice, but voiding patterns differ between *E. coli* strains and methods of infection and can include high volume, low frequency voiding [144] or low volume, high frequency voiding [107,205,206]. 

Many men with histological prostatitis present with a pattern of prostatic infiltrate consistent with prostate autoimmunity [207,208,209], an observation co-opted for the design of mouse models. The prostate ovalbumin expressing transgenic-3 mouse expresses ovalbumin under the control of the androgen-responsive probasin promoter [210,211]. Autologous splenocytes are activated in vitro and transplanted to drive T-cell mediated prostate autoimmunity and inflammation [207]. While the pattern of inflammation and mechanisms of cell proliferation have been carefully studied in this mouse, the urinary physiology phenotype is not well characterized. The experimental autoimmune prostatitis mouse model involves repeated intradermal injections of rat prostate homogenate into mice to drive a T-cell based autoimmune reaction that has been used to examine mechanisms of male LUTD and chronic pelvic pain [212,213].

A non-bacterial mouse model of prostate inflammation was created based on observations that IL-1 beta abundance increases after intraprostatic injection of noxious agents or uropathogenic *E. coli* infection [214,215,216,217] and that Prostatic IL-1 beta abundance is elevated in humans with histological BPH and correlates with LUTS and chronic pelvic pain [218,219,220,221,222]. This mouse model utilizes the Tet-On system which induces expression of a gene in the presence of doxycycline and is tunable, with stronger transgene expression with doxycycline dose [223]. A double transgene of Hoxb13-rTA transgene and a TetO-IL1 beta responder is used to drive IL-1 beta in prostatic epithelial cells [223,224]. The urinary metabolomic proteomic signatures of this mouse have been described, but the urinary physiology phenotype remains to be determined [224,225].

A recent mouse model of prostate inflammation is based off observations that prostate secretory proteins are leaked into prostate stroma of some men with LUTD and accompanied with patchy loss of the adherens junction protein e-cadherin, suggesting a loss of prostate barrier function [226]. Genetic depletion of e-cadherin in mouse prostate epithelium increases prostate mass and cell proliferation, thickens prostate stroma, and increases voiding frequency while reducing voided urine volume, and increases spontaneous bladder contractions [227].

### 4.3. Mouse Models of Partial Bladder Outlet Obstruction (pBOO)

Surgical approaches were first used to model pBOO in male mice. One approach involves a retropubic incision to apply and cinch a suture or metal ring around the bladder neck or pelvic urethra to drive bladder compensation and overactive bladder, and later bladder decompensation, detrusor underactivity, fibrosis, and loss of muscle mass [228,229,230,231]. The mouse model has been essential for recognizing new druggable pathways for restoring function to the decompensated bladder [232].

Treatment with exogenous androgens combined with estrogens is a non-surgical method to drive BOO in mice. Mice are given slow-release implants of androgen (testosterone or dihydrotestosterone) in combination with slow-release implants of estrogen (17beta-estradiol or diethylstilbesterol). The combination of androgen plus estrogen is necessary for prostate gland maintenance, as estrogens delivered to male mice in the absence of androgens disrupts hypothalamic/pituitary/gonadal signaling and cause prostate gland atrophy [128]. Genetically engineered mice that overexpress aromatase are also used to recapitulate the endocrine environment of advancing age [129,233]. Male mice treated with androgen and estrogen develop progressive LUTD, with evidence of disease processes (increased bladder weight as evidence of hypertrophy/compensation for BOO) occurring as early as two weeks after treatment [124]. Sustained exposure to exogenous androgens and estrogens elicits a variety of changes to the male lower urinary tract of multiple species, including prostatic hypertrophy and inflammation, urethral narrowing and abnormal urethral muscle tone, urinary dysfunction with progressive onset, bladder overactivity and eventual decompensation [124,127,128,129,130,131,133,234,235,236,237,238]. Estrogen receptor activation is a key driver of urinary dysfunction, as exogenous estradiol given to male mice drives urinary retention in the absence of exogenous testosterone [239] and estrogen receptor 1 is required for urinary retention and voiding dysfunction from exogenous testosterone and 17beta-estradiol in mice [237]. An important consideration when exogenous androgen and estrogen are used to drive male LUTD, especially when incorporating genetic changes to identify mechanisms, is that hormone responsiveness, disease onset, progression and severity are influenced by genetic background and mouse strain [234]. The delivery system of exogenous androgens and estrogens should be considered if using hormones to drive LUTD for a toxicology study. Compressed pellets of androgens and estrogens can be crushed when animals are restrained for chemical exposure (injection) [21]. Silastic capsule preparations of androgens and estrogens are more durable [240]. 

### 4.4. Mouse Models of Overactive Bladder (OAB)

OAB can be induced by ischemic injury [24,93,241]. A balloon catheter is passed through the iliac artery and inflated, then withdrawn to cause endothelial damage [241]. This injury is combined with a cholesterol enriched diet to cause bladder arterial occlusions and chronic bladder ischemia [206]. This model results in increased voiding frequency but decreased voided volume, and more frequent non-voiding bladder contractions in rats [241]. 

OAB can also be induced by the introduction of noxious stimuli (acetic acid, hydrochloric acid, and others) into the bladder [242,243]. Chemical induced OAB decreases the inter-voiding interval of anesthetized mice, reduces bladder capacity, and sensitizes afferent nerves [243,244,245,246].

### 4.5. Mouse Models of Detrusor Sphincter Dyssynergia (DSD)

The most common approach to evoke detrusor sphincter dyssynergia is to induce spinal cord injury under anesthesia [247]. Urine must be manually expressed at least three times per day until the micturition reflexes recover (10–14 days), then once per day [247]. Cystometry profiling of injured mice reveals increased activity of the external urethral sphincter coupled with increased urethral pressure and voiding pressure, increased frequency and magnitude of non-voiding contractions, and increased bladder capacity [247,248]. 

## 5. Conclusions

Lower urinary physiology is extremely complex, shaped by contributions from the urethra, prostate, bladder, ascending and descending neural pathways, and the brain. Despite an extremely high prevalence of male LUTD and devastating impacts on society, LUTD mechanisms and factors that influence LUTD severity are poorly understood. Environmental contributions to LUTD remain almost completely unexamined. We provided this overview of male lower urinary tract anatomy, physiology, and cell biology, described known disease mechanisms, and highlighted knowledge gaps that require additional research to direct new attention from toxicologists and environmental health specialists to this widespread disease. We detailed examples of environmental chemicals that perturb urinary tract function and described mouse models of LUTD with the intention that public health specialists, epidemiologists and toxicologists will consider LUTD research in toxicity assessments. Future risk mitigation strategies will likely be critical to reducing the burden and severity of LUTD in aging adults. 

## Figures and Tables

**Table 1:** Definitions of terms used to describe anatomical and physiological disorders of the male lower urinary tract.

Acronym	Term	Definition
BPE	Benign Prostatic Enlargement	Non-malignant enlargement of the prostate, defined by imaging or digital rectal exam, and usually caused by BPH.
BPH	Benign Prostatic Hyperplasia	Histologically defined benign growth within the prostate. In humans, the growth pattern is nodular and can be primarily epithelial, stromal or mixed patterns of hyperplasia. BPH is often responsible for BPE.
BPO	Benign Prostatic Obstruction	BOO secondary to BPE.
BOO	Bladder Outlet Obstruction	Blockage of urine passage from an obstruction at the base of the bladder or bladder neck.
	Clinical Prostatitis	A spectrum of conditions characterized by differing degrees of inflammation, bacterial and abacterial, of the prostate, genitourinary tract or pelvis and may not include the prostate.
DO	Detrusor Overactivity	A urodynamic observation characterized by involuntary detrusor contractions during the filling phase that may be spontaneous or provoked.
DSD	Detrsor Spincter Dyssynergia	A disorder where the detrusor muscle contracts while the urethral and/or periurethral sphincter is involuntarily contracted and closed, resulting in bladder outlet obstruction.
	Histological Prostatitis	Prostate inflammation detected in a biopsy specimen.
LUTD	Lower Urinary Tract Dysfunction	A detrimental deviation from normal voiding function. Examples include decreased flow rate, increased voiding frequency, increased or decreased sensation associated with filling, an inability to completely void urine, and an inability to store urine until voluntary release.
LUTS	Lower Urinary Tract Symptoms	Patient described symptoms, scored using the international prostate symptom score, the American Urological Association Symptom index, or other indices that may (or may not) include bother.
OAB	Overactive Bladder	Urgency to urinate with or without urge incontinence, and usually associated with increased voiding frequency.
OVD	Obstruction Voiding Disorder	Lower urinary tract dysfunction deriving from an obstruction in the lower urinary tract.
pBOO	Partial Bladder Outlet Obstruction	Partial blockage of urine passage from an obstruction at the base of the bladder or bladder neck.
	Prostatitism	Male LUTD deriving from a prostatic mechanism
	Prostatomegaly	Prostate enlargement from malignant or non-malignant mechanisms.

**Table 2:** Strengths and limitations of methods to evaluate lower urinary tract dysfunction in men and male mice.

Method in Men	Method in Male Mice	Method Description	Strengths and Limitations
Cell and tissue-based calcium flux assays	Cell and tissue-based calcium flux assays	Calcium indicator dyes or genetically encoded calcium sensors are used to measure intracellular calcium concentrations in response to pharmacological agents and electrical field stimuli.	This method has been applied in vitro with human and mouse tissues and cells, and in vivo with mice, penetration can be limited for calcium indicator dyes and genetically encoded sensors are generally limited to mouse tissues.
Cystometry	Cystometry	A catheter is placed in the bladder and the bladder is filled with water or saline while measuring pressures associated with bladder filling and emptying. The catheter can also be used to collect post-void residual urine in the bladder.	Effective at measuring bladder pressure, but catheter is placed retropublicly in mice and transurethrally in humans which can contribute to intraspecies variability. Baseline pattern can vary by strain in mice.
Cystoscopy	Not available	A cystoscope is inserted into the urethra to visualize the lower urinary tract.	Effective in identifying prostatic enlargement, urethral and bladder inflammation, and some urological cancers, but this method is not available for mice.
Histology and immunohistoche-mistry	Histology and immunohistochemistry	Tissues sections are evaluated for BPH, inflammation and collagen accumulation (definitive diagnosis of BPH, histological prostatitis, fibrosis) and can be used to assess LUTD mechanisms.	Effective for assessing anatomical and cellular changes in lower urinary tract tissues and definitive diagnosis for some urological diseases but is invasive and therefore control tissues are difficult to obtain for healthy men for experimental comparisons; definitive identification of cell types requires complex multiplex protocols.
Isometric contractility	Isometric Contractility	Bladder, prostate, or urethral tissue is mounted in saline bath, pharmacological agents or electrical field stimuli are applied and force displacement is measured.	Quantitative and can reveal specific receptor mediated mechanisms of muscle function but is invasive and destructive to tissue (cannot be easily multiplexed with other methods.
Magnetic resonance imaging	Magnetic resonance imaging	Quantifies bladder wall thickness, detrusor and bladder volume, bladder neck angle, urethral length and diameter and prostate volume.	Can identify mechanisms of LUTD (bladder decompensation, BPE), but time consuming and expensive.
Symptom score	Not applicable	Standardized surveys such as the American Urological Association Symptom Index, the International Prostate Symptom Score, LURN, the National Institutes of Health-chronic prostatitis symptom index (NIH-CPSI) and others are used to quantify urinary symptoms and quality of life	Rapid, inexpensive and can be given repeatedly to monitor disease progression or responsiveness to therapy; limited to humans and not applicable for mice.
Ultrasound	Ultrasound	Quantifies bladder volume and wall thickness, urethral lumen diameter and in mice, velocity of urine as it passes through the urethra.	Fast but high-resolution imaging (for mice) requires expensive equipment.
Uroflowmetry	Uroflowmetry	Performed by measuring voided urine flow and volume.	Non-invasive, but requires specialized equipment, and operator experience and cannot distinguish between anatomical (bladder, prostate, or urethra) mechanisms of LUTD.
Voiding diary	Void spot assay	Men use a journal to record urinary void frequency, timing, and use a capture container to record volume; for mice, a filter paper is placed at the bottom of the cage and later illuminated to quantify void spot number, size, and pattern.	Inexpensive, noninvasive, but can vary by day and individual and cannot distinguish mechanism (bladder, urethra, prostate) of voiding dysfunction.

## Data Availability

This paper does not report any data that has not been published elsewhere.
